# Sugar-House Cure for Consumption

**Published:** 1855-01

**Authors:** 


*Sugar-house Cure for Consumption.*—The Boston Medical and Surgical Journal has recently published two or three articles, by Dr. Cartwright, of New Orleans, upon the sugar-house cure for consumptives, detailing the cases of two or three ladies who had been restored from confirmed phthisis thereby. One of these ladies, in a letter to Dr. Cartwright, says that she had been "stethoscoped" by ourself, and declared phthisical. We have no recollection of the case but do not doubt the accuracy of the statement—or of the diagnosis.

The question of interest in this matter is whether the sugar-house vapor is really an important curative means in consumption, or whether its virtues have not been a good deal exaggerated in the wordy and stilted articles which have recommended it.

On this point we give the following testimony from an esteemed correspondent in New Orleans, in whose judgment, learning, and *reliability*, we have the highest confidence. He says:

"The most reliable physicians, *in the country*, where alone the sugar-houses are, inform me that the whole of it is ridiculous, and that the negroes that constantly work in them are as much or more subject to pulmonary diseases than the others." H.

---

The Philadelphia Medical Examiner for December, has a long review of the trial of Dr. Beale, a dentist of that city, for an alleged violation of a female patient while under the influence of ether. Dr. Hartshorne, in this paper, gives the subject a very thorough analysis. Most of our readers are doubtless familiar with the evidence in this case, and we only mention it to record our personal conviction that the verdict of the jury was entirely unauthorized by the evidence. Physicians familiar with the salacious actions occasionally exhibited in partial anæsthesia and the erotic dreams which ether often occasions, will be loth to convict a hitherto respectable man of rape where the only evidence is that given by the female herself of her sensations during the continuance of the effects of the ether. H.

